# Cellulose Nanofibrils as a Damping Material for the Production of Highly Crystalline Nanosized Zeolite Y via Ball Milling

**DOI:** 10.3390/ma15062258

**Published:** 2022-03-18

**Authors:** Haya Nassrullah, Shaheen Fatima Anis, Boor Singh Lalia, Raed Hashaikeh

**Affiliations:** 1 NYUAD Water Research Center, New York University Abu Dhabi, Abu Dhabi P.O. Box 129188, United Arab Emirates; hfn2004@nyu.edu (H.N.); sfa5@nyu.edu (S.F.A.); bl83@nyu.edu (B.S.L.); 2 Chemical and Biomolecular Engineering Department, Tandon School of Engineering, New York University, Brooklyn, NY 11201, USA

**Keywords:** ball milling, zeolite Y, nanozeolite, cellulose nanofibrils, crystallinity, dye adsorption

## Abstract

Nanosized zeolite Y is used in various applications from catalysis in petroleum refining to nanofillers in water treatment membranes. Ball milling is a potential and fast technique to decrease the particle size of zeolite Y to the nano range. However, this technique is associated with a significant loss of crystallinity. Therefore, in this study, we investigate the effect of adding biodegradable and recyclable cellulose nanofibrils (CNFs) to zeolite Y in a wet ball milling approach. CNFs are added to shield the zeolite Y particles from harsh milling conditions due to their high surface area, mechanical strength, and water gel-like format. Different zeolite Y to CNFs ratios were studied and compared to optimize the ball milling process. The results showed that the optimal zeolite Y to CNFs ratio is 1:1 to produce a median particle size diameter of 100 nm and crystallinity index of 32%. The size reduction process provided accessibility to the zeolite pores and as a result increased their adsorption capacity. The adsorption capacity of ball-milled particles for methylene blue increased to 29.26 mg/g compared to 10.66 mg/g of the pristine Zeolite. These results demonstrate the potential of using CNF in protecting zeolite Y particles and possibly other micro particles during ball milling.

## 1. Introduction

Zeolites are crystalline aluminosilicate materials with a negatively charged framework that have been used in multifarious applications in the industry [1,2]. Among the different types of zeolites, zeolite Y is significantly important since it is utilized for adsorption, separation, and catalysis, among others [3,4,5,6]. The performance of zeolite Y in various applications depends on the crystal size. For instance, Taufiqurrahmi et al. [7] reported that nanocrystalline zeolite Y yielded higher reactant conversion and enhanced the desired product selectivity compared to microcrystalline zeolite Y. In addition, it has been proven that the performance of micro-zeolite in organic dye and heavy metal ions adsorption is limited by the sole presence of micropores in the zeolite structure, which constrains mass transfer [8]. Better performance in catalysis and adsorption applications can be achieved by utilizing nanozeolites. This is mainly related to the larger external surface area, shorter diffusion path lengths, and higher accessibility to active sites in the case of the nanozeolites compared to micro-sized zeolites [2,9].

Recently, nanozeolite Y was utilized as a nanofiller to improve the performance of membranes used for wastewater treatment and desalination. Anis et al. [10] showed that the incorporation of 0.4 wt% of nanozeolite Y in polysulfone (PSf) ultrafiltration membrane resulted in enhanced performance. Compared to the pristine PSf membrane, the nanocomposite membrane exhibited higher average flux and dye rejection by 75% and 66%, respectively. In another study [11], poly (vinyl) alcohol (PVA)-networked cellulose (NC) membranes with nanozeolite Y were prepared for desalination. It was found that the impregnation of 0.5 wt% nanozeolite Y into PVA-NC membrane caused a 34.2% improvement in flux compared to pure PVA-NC membrane. In addition, the salt rejection was not compromised as it was 98.9% for PVA-NC and 99.52% for PVA-NC-nanozeolite Y. Flux enhancement in these studies was attributed to the nonporous 3-D channels in nanozeolite Y which preferentially allow the passage of fresh water due to their intrinsic hydrophilicity. Moreover, higher salt and dye rejections were achieved as a result of size exclusion through the pores and electrostatic interactions. The results showed that with the incorporation of nanozeolite Y in water treatment membranes, the tradeoff between water flux and pollutants rejection can be addressed.

Nanozeolite Y is not available commercially; therefore in small-scale laboratory setups, nanozeolite Y is produced via hydrothermal synthesis. The silicon to aluminum (Si: Al) ratio of nanozeolite Y synthesized by the hydrothermal process is much lower compared to commercial micro-sized zeolite Y. The high silicon content in the nanozeolite Y is essential in improving the stability of these nanoparticles to endure the tough conditions of hydrocracking processes [12,13]. In addition, the complicated nature of hydrothermal synthesis, long time requirement, and limitations created an opportunity for alternative methods to produce nanozeolite. Ball milling of micro-zeolite Y under dry or wet conditions can be used to obtain nanozeolite Y with a high Si: Al ratio. However, reducing the particle size via ball milling is often associated with the destruction of the zeolite external structure and loss of crystallinity. The ball milling media is a key factor which affects changes in zeolite structure and percentage of crystallinity loss. Akçay et al. [14] investigated the effect of wet ball milling on the crystallinity of zeolite Y and compared their results to dry ball milling from previous studies [15,16]. They concluded that particles prepared by wet ball milling have higher crystallinities than those obtained by dry ball milling. This is because, in wet ball milling, the presence of water minimizes the increase in temperature and leads to partial recrystallization of the amorphized regions. Further details on the effect of changing wet ball milling process parameters on the size and crystallinity of the zeolite Y particles were provided by Saepurahman and Hashaikeh [17]. By optimizing the process parameters, they were able to produce nanozeolite Y with an average diameter of 100 nm, a crystallinity index of 20%, and a surface area of 465 m^2^/g. In a subsequent study [18], carbon nanostructure (CNS) was utilized as a damping material for ball milling of micro-zeolite Y to protect the particles from harsh milling conditions and preserve the structure. Although the resulting particles had high crystallinity and surface area, the particle size distribution was wide. Moreover, CNS is a toxic non-biodegradable material by which its release to the environment can cause potential threats to the health of humans and animals [19,20].

Eco-friendly and biodegradable cellulose nanofibrils (CNFs) can be used as an alternative to CNS in preparing nanosized zeolite Y [21,22,23]. Due to its high surface area and mechanical strength as well as gel-like behavior in water, CNF can act as a shield to protect the micro-zeolite Y particles from harsh ball milling conditions [24]. Hence, a further reduction in the particle size can be achieved without significantly compromising particle crystallinity. Therefore, in this study, we examined the effect of using CNF as a damping material in the ball milling of micro-zeolite Y to produce nanosized zeolite Y with high crystallinity. Zeolite Y nanoparticles were characterized in terms of particle size, crystallinity, structure, and surface area using scanning electron microscopy (SEM), transmission electron microscopy (TEM), Brunauer–Emmett–Teller (BET), among others. Lastly, to confirm that the proposed ball milling procedure with CNFs addition did not impact the properties of zeolite Y, the nanoparticles were tested for methylene blue adsorption and their performance was compared to pristine micro-zeolite Y.

## 2. Materials and Methods

### 2.1. Materials

Zeolite Y (CBV 720, SiO_2_/Al_2_O_3_ mole ratio = 30) was supplied by Zeolyst International (Conshohocken, PA, USA) Cellulose nanofibrils (CNFs; 3 wt.% in water, C_x_(H_2_O)_y_) were acquired from Cellulose Lab (Fredericton, NB, Canada). Methylene blue (molecular weight = 319.85 g/mol) and ethanol (C_2_H_5_OH; 99.9%) were purchased from Sigma-Aldrich (St. Louis, MS, USA). All materials and chemicals were used as received.

### 2.2. Ball Milling Procedure

**Figure 1:**
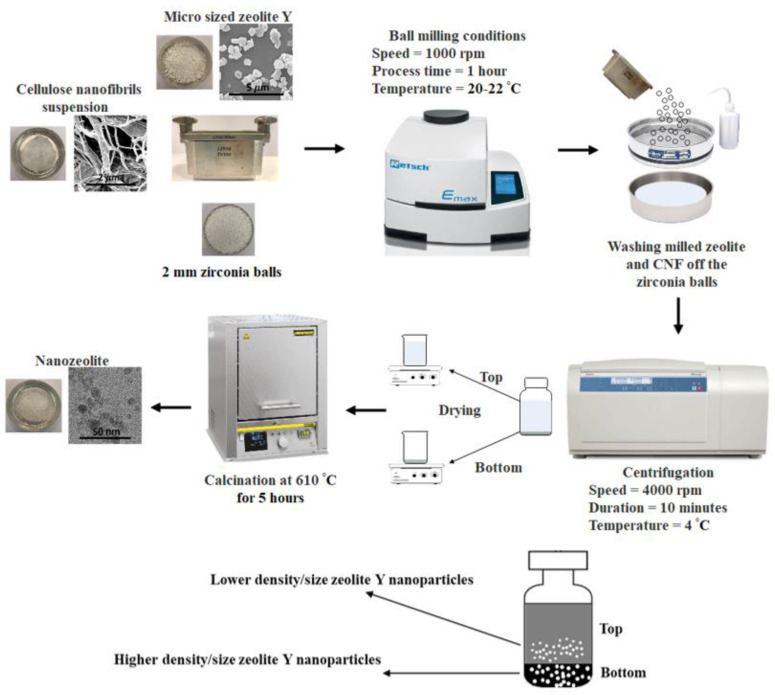
Schematic representation of the procedure followed to prepare nanozeolite Y.

Ball milled zeolites were obtained following the procedure shown in Figure 1. Initially, a mixture of micro-zeolite Y and CNF suspension was placed in a 125 mL zirconia grinding jar with 245 g of 2 mm zirconia balls. Next, the mixture was ball milled at a speed of 1000 rpm for 30 min. High energy milling was performed using an E_max_ ball mill (Retsch, Germany). Throughout the ball milling operation, the temperature was maintained at 20–22 °C using an internal water cooling system as well as an external LT eco-cool Refrigerated Circulating Baths (Grant Instruments) fixed at 10 °C. Furthermore, to avoid overheating the sample, the ball milling machine was paused for 1 min after every 1 min of grinding. Therefore, the total running time was one hour (a total of 30 min of ball milling and 30 min of regular pauses). The milling time was chosen based on a previous study by Saepurahman and Hashaikeh [17]. In their study, ball milling with zirconia balls for 90 min led to severe contamination with zirconia debris. Therefore, 30 min of operation were recommended to avoid such contamination. Different ratios of zeolite Y to CNF (7:3, 6:4, and 1:1) were investigated while keeping the zeolite Y mass constant throughout the study (1 g). After completing each ball milling experiment, the ground mixture of zeolite Y and CNF was washed off from the zirconia balls with deionized water and separated from the balls using a sieve. The suspension of zeolite Y and CNF in water was centrifuged at 4000 rpm for 10 min at 4 ^o^C using the Heraeus Multifuge X3R centrifuge machine (ThermoFisher Scientific, Waltham, MA, USA). Top and bottom products were collected from the centrifuge bottles and dried on a hot plate at approximately 100 °C overnight. To remove CNF, the dried samples were calcined at 610 °C for 5 h using LHTC 08/16 furnace (Nabertherm, Germany). For comparison, a control sample was also prepared by ball milling a mixture of zeolite Y and water without the use of CNF. The calcined and centrifuged ball-milled samples were labeled with the letter T or B to refer to the top or bottom products (see illustration in Figure 1), respectively, followed by the zeolite Y to CNF ratio. For example, the bottom ball-milled sample with a zeolite Y to CNF ratio of 1:1 is labeled as B11. More details are summarized in Table 1 below.

### 2.3. Characterization

High-resolution images of the pristine zeolite Y and ball-milled zeolite Y particles were obtained using FEI Quanta 450 Field Emission Scanning Electron Microscopy (SEM) (ThermoFisher Scientific, Waltham, MA, USA) operating at 30 kV. Sample preparation involved dispersion of a few milligrams of particles in ethanol using a Q700 Ultrasonic Processor (Qsonica, Newtown, CT, USA). Next, a pipet was used to place a drop of the sonicated samples on silicon wafers. After the evaporation of ethanol and before conducting the SEM analysis, the samples were coated with an ultrathin layer of gold using 108 Auto Sputter Coater (Ted Pella, Redding, CA, USA) to reduce charging and improve the secondary electrons signal. Particle size distribution was determined by measuring the size of 100 particles using SEM (FEG Quanta 450, FEI). The results were used to generate the particle size distribution graphs and to identify the median value (D50). The surface morphology of optimal ball-milled particles was also studied using the FEI Talos F200X transmission electron microscope (TEM) (ThermoFisher Scientific, Waltham, MA, USA).

Thermal gravimetric analysis (TGA) of CNF and a mixture of ball-milled zeolite with CNF was carried out using TG 209 F3 Tarsus (NETZSCH, Germany). A heating rate of 30 °C/min from room temperature to 650 °C under a nitrogen flow of 20 mL/min was used in the analysis. A Panalytical Empyrean diffractometer was used to determine the phase crystallinities of the samples. Powder diffractograms were obtained by scanning the samples between 10° and 40° at a scan rate of 3° per min. The total area under peaks at 15.7°, 18.7°, 23.7°, 27.1°, 30.8°, 31.5°, and 34.2° was computed after background subtraction for all samples. The calculated area of the ball-milled samples was divided by the area obtained from the pristine zeolite Y diffractogram to find the crystallinity index (%). The pristine zeolite Y was assumed to have a crystallinity index of 100% as the reference for comparison with the ball-milled particles. A Nicolet iS 5 Fourier transform infrared (FT-IR) spectrometer (ThermoFisher Scientific, Waltham, MA, USA) was used to compare the chemistry of micro-zeolite with the ball-milled zeolites. Each sample was scanned 16 times in the wavelength range of 4000 to 430^−1^.

Brunauer-Emmett-Teller (BET) analysis was performed using NOVA e-4200 surface area and pore size analyzer (Quantachrome Instruments, Boynton Beach, FL, USA). Preceding BET analysis, pristine zeolite Y and optimal ball-milled samples were dried under vacuum at 350 °C. Data from the nitrogen adsorption curves at a relative pressure (*P/P_o_*) of 0.05 to 0.35 were used to determine the micropore surface area of particles (S_MP_). t-plots were used to obtain the external surface area (S_t_) and micropore volume (V_MP_) of particles by analyzing the plots at a thickness between 3.5 and 5 Å. A t-plot is a plot of the volume of gas adsorbed versus the statistical thickness of an adsorbed film (*t*). The De Beor equation was used to calculate statistical thickness (*t*) as a function of the relative pressure (*P/P_o_*) as follows:

(1) $$t\;\left(\text{Å}\right)={\left[\frac{13.99}{\log\;\left(\frac{P_{o}}{P}\right)+0.034}\right]}^{1/2}$$

The following equation was used to calculate the total pore volume (*V*) in cm^3^/g:

(2) $$V=\frac{P_{a}\;V_{abs\;}V_{m}}{R\;T}$$

where *P_a_* is the ambient pressure (1 atm), *V_abs_* is the amount of vapor adsorbed at *P/Po*  ≈ 1 in cm^3^/g, V_m_ is the molar volume of liquid nitrogen (34.7 cm^3^/mol), T is the ambient temperature (293 K), and *R* is the ideal gas constant (82.05 cm^3^.atm/mol.K).

### 2.4. Dye Adsorption Experiment

The dye adsorption capacity of the ball-milled zeolite particles was examined using a 20 ppm solution of methylene blue dye. Initially, 0.05 g of each sample was added to 100 mL of the methylene blue solution with a pH of 7.5. Next, the flasks were shaken at 125 rpm and 25 °C for 1 h. After that, the absorbance of methylene blue in the supernatant was measured at 664 nm using a UV-3100PC spectrophotometer (VWR, Radnor, PA, USA). Adsorption capacity (*Q*) in mg of dye adsorbed per gram of zeolite powder was calculated as follows:

(3) $$Q=\frac{\left(c_{o}-c\right)\times V}{m}$$

where, *c_o_* is the initial concentration of the dye solution (ppm), *c* is the dye concentration after adsorption, *V* is the solution volume (L), and *m* is the adsorbent mass (g).

## 3. Results and Discussion

### 3.1. Particle Size Distribution and XRD Analysis

**Figure 2:**
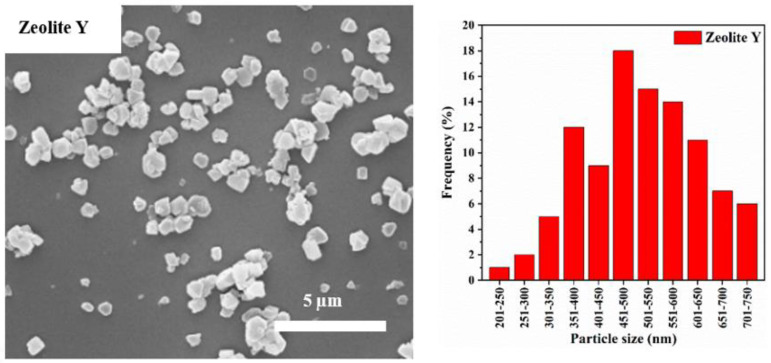
SEM images (**left**) and particle size distribution (**right**) of commercial zeolite Y particles.

**Figure 3:**
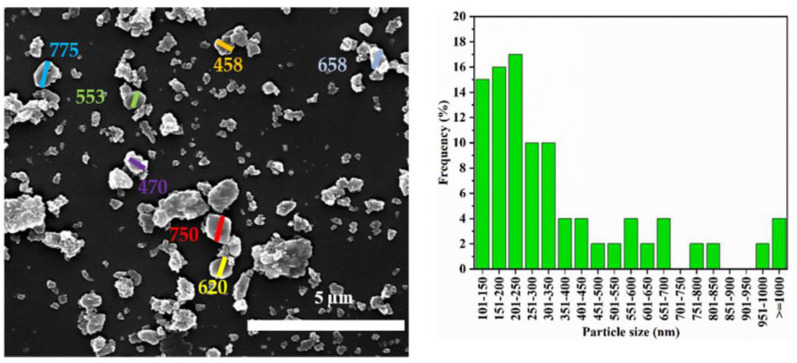
SEM image (**left**) and particles size distribution (**right**) of ball-milled zeolite Y without CNF.

The SEM image and particle size distribution of the pristine zeolite Y particles are shown in Figure 2, where a wide size distribution of the zeolite Y particles can be seen. The particles size ranges from 201 to 750 nm where 50% of the particles have a diameter greater than 547 nm. The SEM image and particle size distribution of the ball-milled zeolite Y particles without CNF addition are also shown in Figure 3. It can be seen that the particle size of zeolite Y is significantly reduced after ball milling without the presence of CNF. However, this also leads to the formation of larger/flake-shaped particles which are bigger than parent zeolite Y. It is widely recognized that ball milling can lead to the formation of larger clusters/flakes of particles as a result of the collision and rolling actions among the powder and balls used as milling media [17,25,26]. To avoid such undesirable clusters/flakes, longer ball milling duration or smaller balls can be used. However, both methods can result in severe destruction of the nanomaterials structure [14,17]. Alternatively, the use of damping material during the ball milling process can reduce this effect significantly without compromising the nanomaterials’ physical/chemical properties.

**Figure 4:**
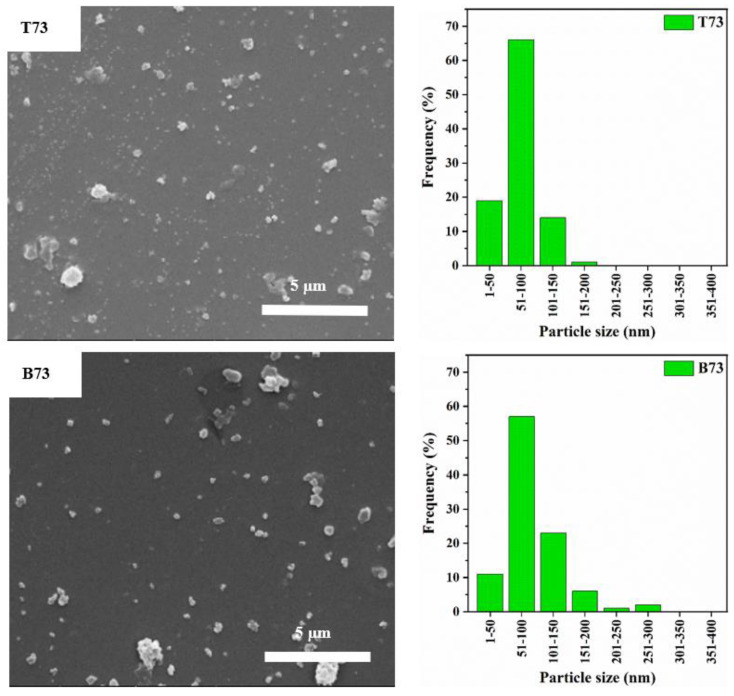
Confirmed the effective role of CNF in reducing the particle size of zeolite Y in ball milling. The use of CNF as damping/protection material created a larger area of contact for the zeolite Y particles during the ball milling which significantly improved the effectiveness of the ball milling process and prevented the formation of flakes/particles clusters.

**Figure 5:**
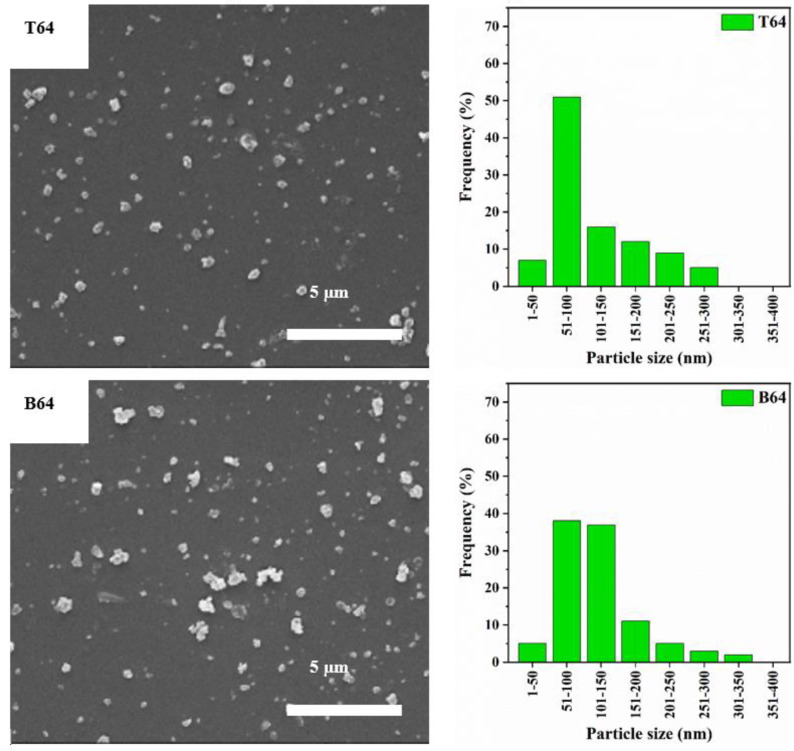
SEM images (**left**) and particles size distribution (**right**) of the ball-milled sample of zeolite Y: CNF = 6:4.

**Figure 6:**
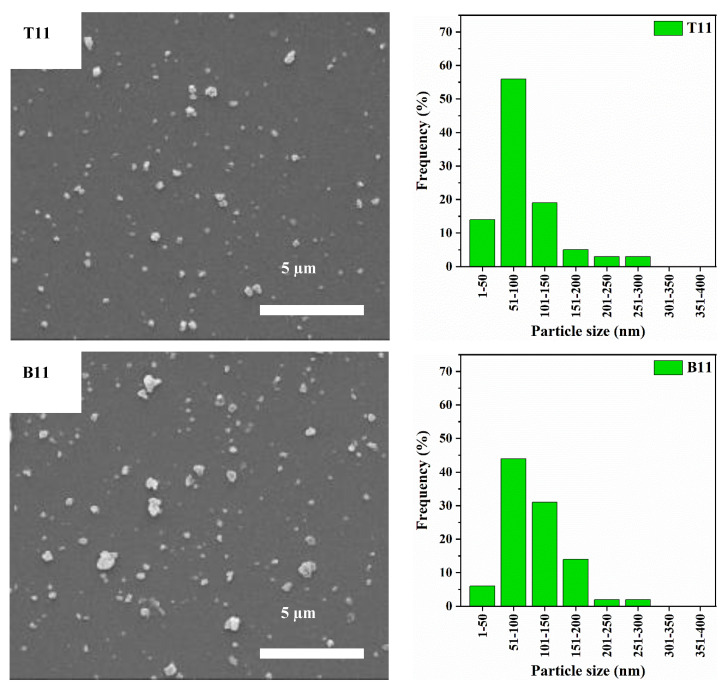
SEM images (**left**) and particle size distribution (**right**) of the ball-milled sample of zeolite Y: CNF = 1:1.

The SEM images and particle size distribution of the ball-milled zeolite Y with different amounts of CNF are shown in Figure 4, Figure 5 and Figure 6. Initially, a sample of zeolite Y to CNF of 7:3 ratio was prepared and ball-milled. This ratio was chosen based on the optimum conditions identified by Zhuman et al. [18] in a previous study where CNS was used as the damping material. The SEM images in Figure 4 reveal a significant reduction in the zeolite Y particles size when compared to the ball-milled parent zeolite Y particles. Moreover, these SEM images show that particles in the top part of the centrifuged sample (T73) are smaller than particles in the bottom part (B73) (Figure 4 left). For instance, 66% of the particles size is in the range of 51–100 nm in the T73 sample compared to 56.6% in the B73 sample. Similarly, 19% of the particles in the T73 sample are in the 1–50 nm range compared to only 10.8% in the B73 sample. The D50 values for T73 and B73 were found to be 69 and 80 nm, respectively. This indicates that the frequency of nanoparticles with a diameter less than or equal to 69 nm in the top product (T73) is higher than the frequency of these nanoparticles in the bottom product (B73). This is mainly related to the fact that larger particles migrate towards the bottom of the bottle during the centrifugation, creating a higher density bottom supernatant [18]. It is important to note that sample centrifugation was carried out to obtain a narrow particle size distribution.

**Figure 7:**
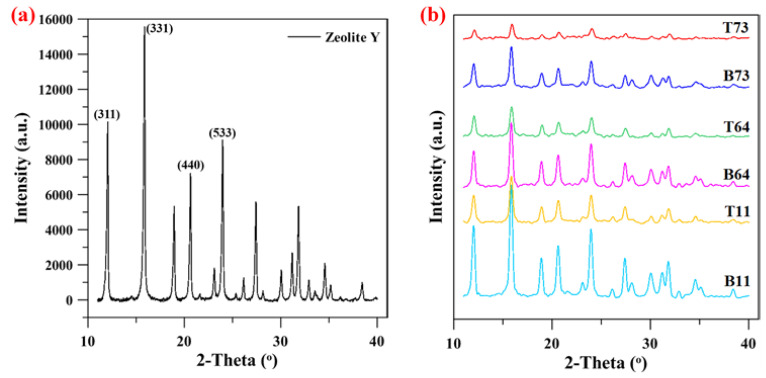
XRD graph of (**a**) Pristine/commercial zeolite Y and (**b**) Ball-milled samples.

To further understand the effect of adding CNF to the ball milling mixture, the ratio of zeolite Y to CNF in the ball milling sample was varied. Comparison between the different ratios was carried out by characterizing the obtained particles in terms of particle size distribution and crystallinity. Figure 5 left and Figure 6 left show the SEM images of products obtained after ball milling of 6:4 and 1:1 ratios of zeolite Y to CNF, respectively. These images show that the size of the particles in the top product is always smaller than the size of particles in the bottom product which is in agreement with the previous observations in the T73 and B73 samples shown earlier in Figure 4 left. The particle size distributions of the 6:4 and 1:1 ratios samples show that increasing the amount of CNF suspension in the sample causes a decrease in particle size. In other words, the frequency of nanoparticles with a diameter less than or equal to 100 nm decreased in both samples when compared to the T73 and B73 samples. For instance, 43% of the particles in B64 have a particle size less than or equal to 100 nm, whereas in B11 the size of 51% of the particles is below or equal to 100 nm (Figure 5 and Figure 6). However, the percentage of particles with a diameter less than or equal to 100 nm in B73 is 67% which is higher than those in B64 and B11. Nevertheless, such a slight reduction in the percentage of the particle size that is less than 100 nm when the CNF ratio was increased is counterbalanced with the increase in the degree of crystallinity, as shown in Figure 7 and summarized in Table 2.

The parent zeolite Y and the ball-milled samples were characterized using XRD and the results are illustrated in Figure 7. The typical characteristic XRD pattern of the commercial zeolite Y particles with all major peaks at 12.1°, 15.9°, 20.7°, and 24.1° is displayed in Figure 7a. These peaks are attributed to the respective planes of (311), (331), (440), and (533), respectively [27]. Similar peaks are obtained in all the ball-milled samples with different ratios of CNF (Figure 7b). This indicates that the basic faujasite structure of the zeolite Y particles was not affected after ball milling. This observation is also in accord with the work reported by Zhuman et al. on similar zeolite Y particles [18]. Figure 7b shows that the sharpness and intensity of the peaks increase as a higher ratio of CNF to zeolite Y is used. Furthermore, to get a deeper insight into the XRD results, quantitative XRD values were obtained by determining the crystallinity index of all the ball-milled samples as summarized in Table 2. From the table, it can be seen that the samples with higher zeolite to CNF ratio have lower crystallinity index when compared to the samples with higher CNF to zeolite ratios. For instance, the crystallinity index of B73 was only 13% whereas B11 reported a higher crystallinity index of around 32%. The XRD results in Table 2 indicate that the crystallinity index decreased in the following order: B73 < B64 < B11 and T73 < T64 < T11. The reduction of crystallinity as a result of ball milling can be attributed to the destruction of the Si-O-Al and Al-O-Al external framework [28]. The severity of this destruction decreases as a higher amount of CNF was used due to the role of CNF in protecting the zeolite particles from harsh collisions during ball milling.

Using ball milling, Saepurahman and Hashaikeh [17] were able to obtain nanozeolite with a D50 of 100 nm and a crystallinity index of 13% only. In this study, the obtained nanozeolite had a narrow particle size distribution with a D50 < 106 nm and crystallinity index up to 32%. These results proved that the addition of CNF to zeolite before ball milling aids in significantly reducing the particle size while protecting the particles from extreme structural destruction. In addition, it prevents the formation of large clusters of particles or the presence of flake-like structures. Lastly, it is worth mentioning that at each zeolite Y to CNF ratio, characteristic peaks in the XRD pattern of the top particles are broader and less intense than those observed in the XRD pattern of the corresponding bottom particles (Figure 7b). These results were also confirmed by comparing the crystallinity indices of top and bottom particles (Table 2). For example, the T73, T64, and T11 reported crystallinity index values of 5%, 9%, and 12% compared to 13%, 21%, and 32% for the B73, B64, and B11 samples, respectively. The lower crystallinity of the top samples can be related to the presence of a higher amount of smaller particles which were exposed to harsher ball milling. Similar observations were reported in the literature [18].

### 3.2. Thermal Analysis

**Figure 8:**
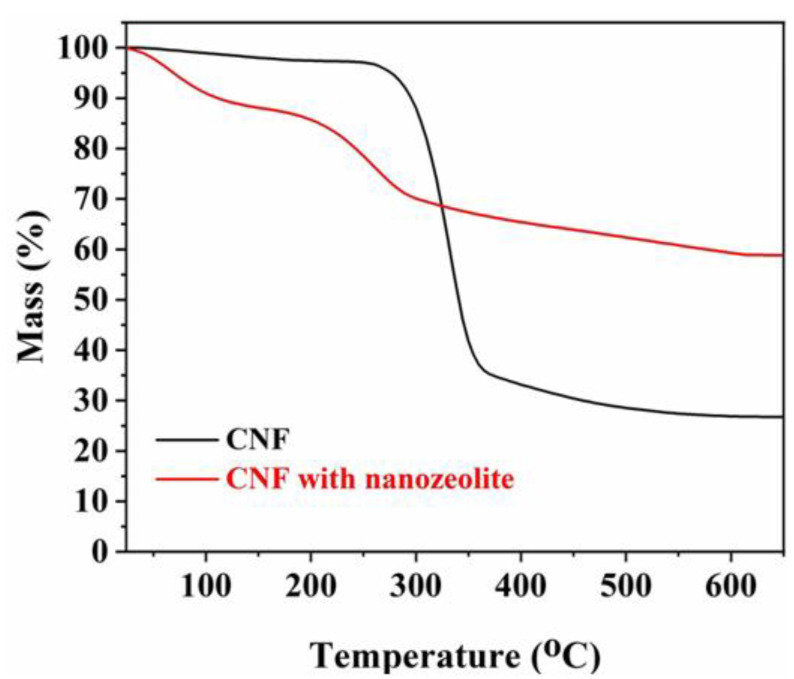
TGA thermograms of CNF and the mixture of ball-milled zeolite with CNF before calcination.

To confirm that the chosen calcination temperature of 610 °C was sufficient to remove all the CNF used, TGA analysis was performed, and the results are displayed in Figure 8. The thermal degradation curve of CNF shown in Figure 8 consists of three decomposition stages. The first decomposition stage below 170 °C is mainly related to the evaporation of the entrapped/adsorbed water molecules on the cellulose fibers. The second stage at around 260 °C is attributed to the degradation of the cellulose structure whereas the last stage above 450 °C is related to the further decomposition of second stage products. Similarly, during the increase in temperature from 35 °C to 170 °C, the mass loss of the ball-milled zeolite with CNF is related to the entrapped water evaporation. On the other hand, the degradation temperature of cellulose structure in CNF/Zeolite Y composite shifted to a lower temperature (200 °C) when compared to the degradation temperature of the CNF alone. This is probably related to the decomposition of oxygen-containing groups in the zeolite Y. Lastly, the TGA curve of both samples showed no further degradation above 600 °C (Figure 8). Therefore, it is safe to say that a temperature of 610 °C was sufficient to remove the CNF used during the ball milling process through the calcination step used after.

### 3.3. Surface Morphology and Functionalities

**Figure 9:**
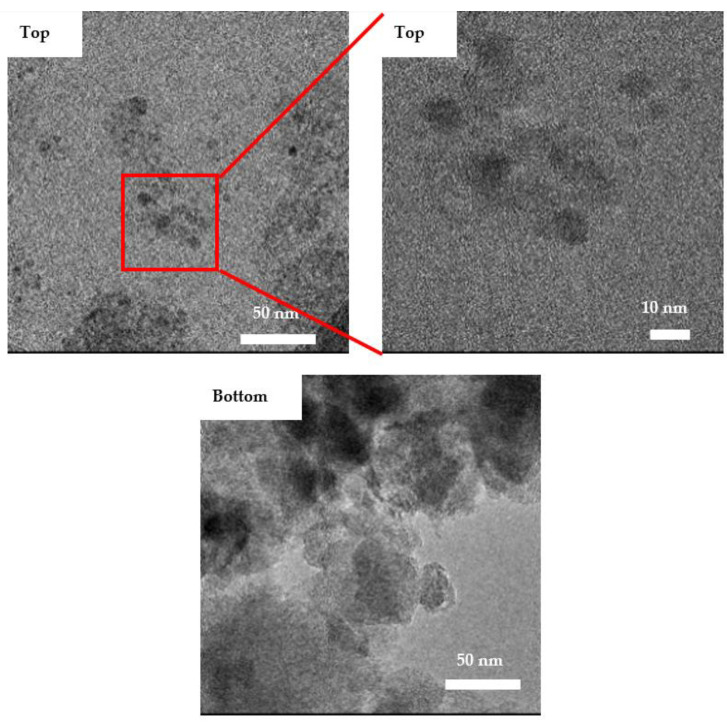
TEM images of the top and bottom products of the ball-milled samples.

To observe the change in zeolite structure as a result of ball milling, the TEM images of the top and bottom products of the ball-milled sample are compared (Figure 9). The ball-milled zeolite particles from the top sample appear to be more uniform with a spherical shape by which the smallest observed particle size is around 5–10 nm. On the other hand, the particles in the bottom sample appear to be more random in shape with larger particles size when compared to the top sample. This indicates that the tendency to form spherical particles increases with the decrease in size. These results are in agreement with the SEM images of the ball-milled samples shown earlier that revealed smaller particles size in the top samples when compared to the bottom samples. For example, the cumulative frequency of particles that are less than or equal to 100 nm in size is 85% in the T73 sample compared to only 67% in the B73 sample (Table 2). Similar observations can be found in the remaining samples.

**Figure 10:**
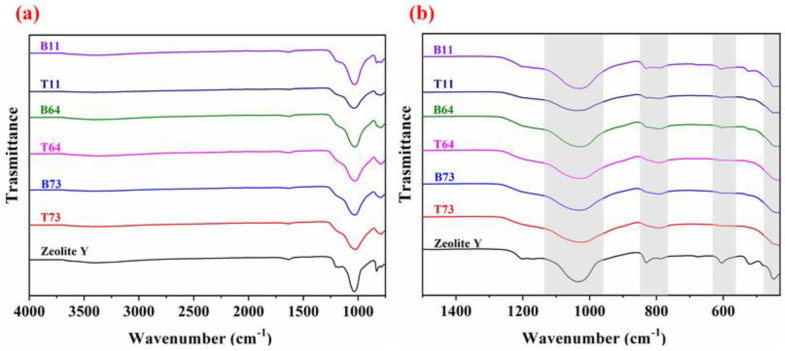
FT-IR spectra of the pristine zeolite Y and ball-milled CNF/zeolites in the wavelength range from (**a**) 4000 cm^−1^ to 600 cm^−1^ and (**b**) 1500 cm^−1^ to 430 cm^−1^.

The FT-IR spectra were obtained for pristine zeolite Y and the ball-milled zeolite samples to examine the functionalities and any possible changes in the samples after the ball milling process (Figure 10). Essentially, the main characteristic FT-IR peaks of zeolite Y are present in the 1300–430 cm^−1^ region (Figure 10b). Bands in this region are attributed to internal vibrations of the TO_4_ tetrahedron (T=Si, Al) and vibrations of external linkages between the tetrahedron. The strong broad band at 1040 cm^−1^ is attributed to the asymmetric stretching vibrations of internal O-T-O in the zeolite Y particles whereas the band at around 1200 cm^−1^ is related to the asymmetric stretching vibrations of the external TO_4_ structure (T = Si, Al) [29,30]. Symmetric stretching of external linkages is evident from the weak bands in the range of 760–850 cm^−1^ [29]. The medium band at 630–570 cm^−1^ is related to the presence of double-ring polyhedral in the secondary building unit [29,30,31]. The next strongest band at 430–480 cm^−1^ is attributed to the structure-insensitive bending modes of T-O [29,30]. Weak bands at 1640 cm^−1^ and around 3400 cm^−1^ are attributed to the proton vibration in the water molecules entrapped in the zeolite structure (Figure 10a). The FT-IR spectra of ball-milled zeolites are similar to the pristine zeolite Y spectrum with minor differences in the intensity of some peaks. For example, the intensities of the bands corresponding to vibrations of external linkages at 760–850 cm^−1^ and 630–570 cm^−1^ decrease after ball milling. This decrease is caused by the partial destruction of the external zeolite framework and a decrease in the overall crystallinity. It is observed that the similarity between the FT-IR spectra of pristine zeolite Y and ball-milled zeolite increases as the crystallinity index of the ball-milled particle increases. This is also related to the amount of CNF in the ball milling mixture because samples with higher CNF contenta yielded particles with a higher crystallinity index. The presence of CNF in the ball milling mixture protects zeolite particles from harsh milling such that the crystallinity and external structure are partially preserved, which is evident from the FT-IR spectra.

### 3.4. BET Analysis

**Figure 11:**
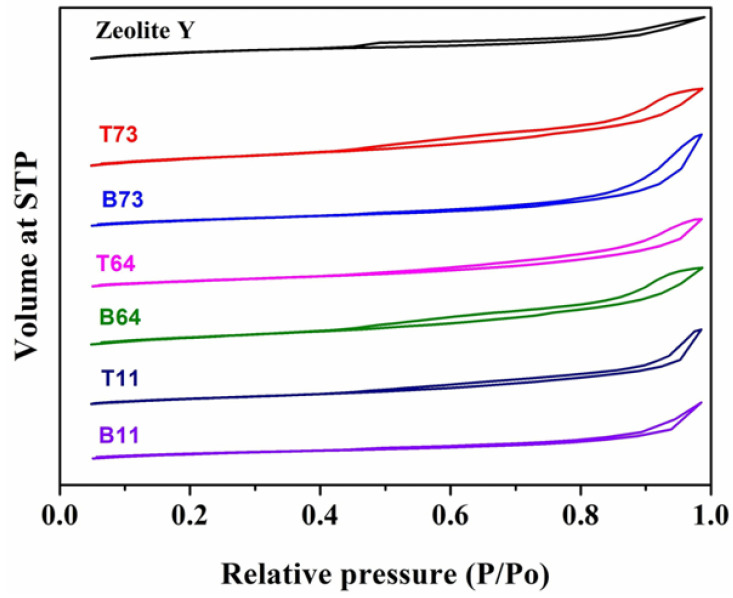
Adsorption-desorption isotherms of nitrogen for parent zeolite Y and ball-milled sample.

Nitrogen adsorption-desorption curves of the original zeolite Y and ball-milled samples are shown in Figure 11. The hysteresis loop of all samples has a starting/closing point from P/Po between 0.4 and 1.0 which suggests the presence of mesopores in the structure. The isotherm hysteresis shape of the ball-milled sample is similar to parent zeolite Y. This indicates that the mesoporous structure of zeolite Y was retained after ball milling. To quantitatively examine the difference between pristine zeolite Y and the ball-milled samples, the external and micropore surface areas as well as total and micropore volumes were determined (Table 3). Ball milling of zeolite particles led to an increase in the external surface area as a result of the decrease in particle size. For instance, the top and bottom ball-milled samples have a higher external surface area compared to pristine zeolite Y (Table 3).

Furthermore, in all ratios of zeolite Y to CNF, the external BET surface area (St) of the top particles is higher than the bottom particles which is attributed to the difference in particle size. This observation is supported by the results in Table 2 that show that D50 of the top particles is lower than D50 of the bottom particles in all of the samples. This indicates that particles in the top product are smaller than particles in the bottom product. Ball milling of micro-zeolite also leads to the depletion of microporosity as a result of reducing the crystalline phase [16]. The results in Table 3 show that at a zeolite Y to CNF ratio of 7:3, BET micropore area (SMP) of the top and bottom products is reduced by 87% and 78%, respectively, when compared to pristine zeolite Y. However, this reduction in the SMP is minimized when the amount of CNF is increased in the sample. For example, in the B11 sample (i.e., highest CNF content), the reduction in the SMP was only 64% when compared to the pristine zeolite Y sample. This is related to the fact that ball-milled samples with a higher CNF to zeolite Y ratio have a higher crystallinity index (Table 2). Therefore, compared to all other ball-milled samples, T73 has the highest reduction in SMP whereas B11 had the lowest reduction in the SMP (Table 3).

The wet ball milling of zeolites affects the micropore volume. According to the results in Table 3, the micropore volume of ball-milled particles is lower than parent zeolite Y. For example, the micropore volume of the pristine zeolite Y decreased from 0.354 cm^3^/g to as low as 0.042 cm^3^/g in the T73 sample. It is also observed that the micropore volume of the bottom particles is higher than the top particles which is mainly related to the crystallinity of the samples (Table 2). In a previous study, Huang et al. showed that the micropore volume increases as the crystallinity becomes higher [15]. Similarly, samples with a lower amount of CNF have a lower micropore volume as their crystallinity index is lower. Overall, the external surface area of all the ball-milled samples increased when compared to the pristine zeolite Y sample.

### 3.5. Dye Adsorption Study

**Figure 12:**
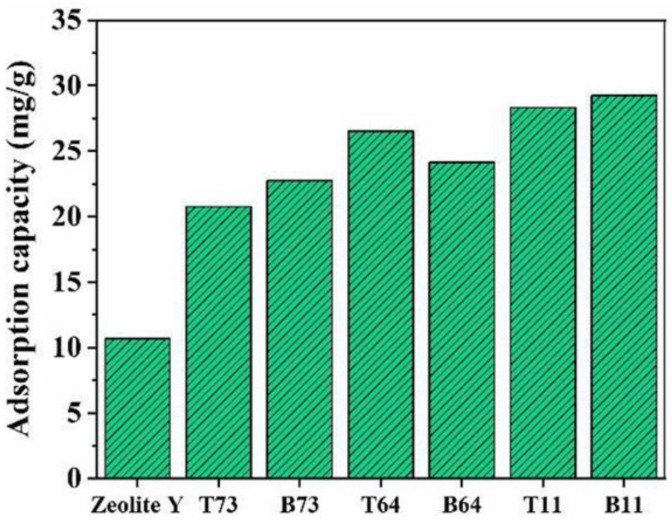
The adsorption capacity of micro and nanozeolites.

Zeolite Y and the ball-milled zeolites were tested for methylene blue dye adsorption. The adsorption performance of these particles was evaluated by determining the adsorption capacity. As shown in Figure 12, pristine zeolite Y particles reveal the lowest adsorption capacity among all the samples (10.66 mg/g). This is related to the dominant presence of micropores which limits the mass transfer of dye molecules such that the interaction between the active sites in zeolite and the dye is significantly hindered [32]. The adsorption of methylene blue by zeolite Y is mainly governed by electrostatic interactions between the cationic dye and the negatively charged zeolite Y [33,34]. On the other hand, nanozeolites obtained by wet ball milling show a higher adsorption capacity of methylene blue compared to pristine zeolite Y. For example, the highest adsorption of 29.26 mg/g was observed in the B11 sample, which is 63.5% higher than the adsorption via the pristine zeolite sample (Figure 12). Similarly, all other ball-milled samples show improved adsorption capacity when compared to the pristine zeolite Y sample. This is attributed to the partial destruction of the external structure of zeolite during ball milling which increases the accessibility to the active sites leading to more probable interaction between the dye and zeolite. Similarly, Anis et al. [3] stated that hydrocracking fibers made from zeolite-Y nanoparticles produced through ball milling had higher acidity compared to fibers made from parent zeolite Y. This indicates that ball-milled nanoparticles have more accessible active sites compared to pristine zeolite Y.

## 4. Conclusions

In this study, CNF was used as a damping material in the wet ball milling of micro-zeolite Y to produce nanosized particles and minimize the loss in crystallinity. Different ratios of zeolite Y to CNF were studied (7:3, 6:4, and 1:1) and characterized. The SEM images showed that the particle size of zeolite Y was significantly reduced by ball milling with CNF as a damping material. The XRD and FT-IR results showed that the main building blocks of the faujasite crystal structure were retained after ball milling. This was confirmed through the appearance of the main characteristic peaks of zeolite Y in the XRD patterns and FT-IR spectra of the ball-milled samples. Moreover, the loss in crystallinity decreased as the amount of CNF in the ball milling mixture increased. This was attributed to the fact that CNF acts as a shield to protect the particles from harsh crushing during the milling process. BET results revealed that the mesoporous structure of zeolite Y was retained after ball milling. Moreover, dye adsorption experiments showed that nanozeolites were more effective in the adsorption of methylene blue compared to micro-zeolite Y due to the greater accessibility to active sites and higher ion-exchange capacity. Based on the characterization results, it was found that the optimal zeolite Y to CNF ratio was 1:1 with a crystallinity index of 32%, D50 of 100 nm, and adsorption capacity of 29.26 mg/g for the bottom product (B11). Overall, the use of CNF as damping material showed great potential in protecting the zeolite Y particles from harsh milling conditions, producing highly crystalline structures with more accessible active sites and lower particle sizes. Thus, CNF can be utilized in ball milling for different micro materials for different applications.

## Figures and Tables

**Table 1:** Summary of the zeolite Y to CNF ratios used in this study.

Label	Zeolite Y: CNF	Product Location *
T73	7:3	Top
B73	7:3	Bottom
T64	6:4	Top
B64	6:4	Bottom
T11	1:1	Top
B11	1:1	Bottom

* With respect to the product location in the centrifuge bottle.

**Table 2:** Summary of crystallinity index, D50 results, and cumulative frequency of particles with diameter <100 nm for commercial zeolite Y and all ball-milled samples.

Label	Crystallinity Index (%)	Median Particle Diameter D50 (nm)	Cumulative Frequency of Particles with Diameter <100 nm (%)
Zeolite Y	100	547	0
T73	5	69	85
B73	13	80	67
T64	9	89	58
B64	21	106	43
T11	12	82	70
B11	32	100	51

**Table 3:** Surface area and pore volume of parent zeolite Y and ball-milled samples.

Sample	Surface Area (m^2^/g)		V_MP_ (cm^3^/g)	V (cm^3^/g)
	St	SMP		
Pristine zeolite Y	91	619	0.354	0.531
T73	238	78	0.042	0.473
B73	148	135	0.074	0.541
T64	178	95	0.053	0.419
B64	105	148	0.081	0.473
T11	180	101	0.059	0.462
B11	99	220	0.122	0.399

## Data Availability

Data available from the author.
